# Cloning, Expression, and Purification of a GDSL-like Lipase/Acylhydrolase from a Native Lipase-Producing Bacterium, *Lactobacillus fermentum*

**DOI:** 10.52547/ibj.26.2.153

**Published:** 2021-12-12

**Authors:** Samira Pirmanesh, Rouha Kasra Kermanshahi, Sara Gharavi, Elahe Mobarak Qamsari

**Affiliations:** 1 Department of Biotechnology, Faculty of Biological Sciences , Alzahra University, Tehran, Iran;; 2 Department of Microbiology, Faculty of Biological Sciences, Alzahra University, Tehran, Iran

**Keywords:** *Escherichia coli*, Gene expression, *Lactobacillus*, Lipase, Phylogeny

## Abstract

**Background:**

Lipase enzymes are of great importance in various industries. Currently, extensive efforts have been focused on exploring new lipase producer microorganism as well as genetic and protein engineering of available lipases to improve their functional features.

**Methods:**

For screening lipase-producing lactobacilli, isolated strains were inoculated onto tributyrin agar plates. Molecular identification of lipase-producing Lactobacilli was performed by sequencing the 16Sr DNA gene, and a phylogenetic tree was constructed. The LAF_RS05195 gene, encoding lipase protein in *L. fermentum* isolates, was identified using specific primers, amplified by PCR (918 bp) and cloned into the pET28a (+) vector. The recombinant proteins were expressed 2, 4, 6, and 12 hours after induction with IPTG and assessed using the SDS-PAGE. Enzymatic activity of the purified recombinant protein was measured at 410 nm in the presence of *ρ-*NPA and *ρ-*NPP.

**Results:**

Among five identified native lipase-producing isolates, one isolate showed 98% similarity with Enterococcus species. The other four isolates indicated 98% similarity to *L. fermentum*. After purification steps with Ni-NTA column, a single protein band of about 34 kDa was detected on SDS- PAGE gel. The enzymatic activity of purified recombinant protein alongside *ρ-*NPA and *ρ-*NPP was measured to be 0.6 U/ml and 0.2 U/ml, respectively.

**Conclusion:**

In the present research, a novel lipase/esterase from *L. fermentum *was cloned and expressed. The novel lipase/esterase has the merit to be further studied due to its substrate specificity.

## INTRODUCTION

Carboxylesterases (EC 3.1.1.1) and triacyl-glycerol lipases (EC 3.1.1.3) are two major types of lipolytic enzymes. The main difference between these two groups of enzymes is based on the type of their substrate. Carboxylesterases hydrolyze esters with short-chain fatty acids, while lipases hydrolyze long-chain triacylglycerols^[^^1^^,^^2^^]^. Many lipase and esterase sequences include the GxSxG pentapeptide motif, in which the active site of serine is located at the conserved pentapeptide center. In a new hydrolytic/lipolytic enzyme subfamily named GDSL hydrolases, the active site of serine is located near the N-terminus^[^^3^^]^. The GDSL hydrolases can bind to different substrates through their flexible active site. Due to these properties, GDSL lipases have potential applications in food, pharmaceutical and detergent industries^[^^3^^]^. 

GDSL hydrolytic enzymes are widely found in microbes and plants ^[^^4^^]^. Lactic acid bacteria are widely used in a variety of fermented milks, cheeses, vegetables, fruits, meats, and fish products^[^^5^^]^. Although there is an extensive literature on the cloning and expression of bacterial GDSL lipase genes, little information is available concerning GDSL lipase of Lactobacillus spp., probably due to relatively lower rates of lipase production in such microorganisms^[^^6^^,^^7^^]^. Moreover, failure in replication of results, undesirable side effects, and the complexity of purification procedure render lipase extraction from culture supernatants difficult. Understanding the lipases catalytic mechanism and binding sites, along with overexpression in suitable hosts, are among the advantages of the recombinant DNA technology^[^^8^^-^^10^^]^. 

*L. fermentum* IFO_3956 has generally various lipolytic enzymes encoding genes, namely LAF_RS05195, which is responsible for GDSL-like Lipase/Acylhydrolase, LAF_RS05200 for lipase and LAF_RS05205 for esterase protein^[^^11^^]^. The aim of this research was to clone, express and purify a new GDSL-like Lipase/Acylhydrolase from a native lipase-producing bacterium, *L. *fermentum, by using LAF_RS05195 gene from *L. fermentum *IFO_3956.

## MATERIALS AND METHODS

**DNA manipulation tools and chemical reagents**

*Eco*RI, *Sac*I, and T4 DNA ligase were obtained from Fermentas (Germany), Taq polymerase from CinnaGen (Iran), Ni-NTA resin from BIO-RAD (USA), Ron's Gel Extraction Kit from Bioneer (Korea), and EZ-10 Spin Column PCR Purification Kit from Bio Basic Inc. (Canada). The *ρ*-NPA, *ρ*-NPP, and IPTG were acquired from Sigma–Aldrich (St. Louis, USA).

**Strains, plasmids, and growth conditions**

*E. coli *DH5α strain was used as a host for subcloning and plasmid propagation. Also, *E. coli *BL21 (DE3) was utilized as the specific expression host. The pET28a(+) vector (Novagen, Amsterdam, the Netherlands), containing kanamycin resistance gene, was employed as the expression vector. In order to identify the recombinant strains, 50 µg/ml of kanamycin was added to LB medium. Solution of 1 mM of IPTG was used as an inducer for protein expression, and the strains were grown at 37 °C.

**Isolation of potential lipase-producing lactobacilli from native dairy products**

In our previous research, 25 acid tolerant isolates of Lactobacillus spp. were screened from different traditional dairy products^[^^12^^]^. All the isolates were inoculated onto tributyrin agar plates and incubated at 37 °C for 24 hours. Direct observation of clear zones around colonies suggested lipolytic activity^[^^13^^]^. Colonies having clear zones were selected, purified on tributyrin agar plates and transferred to agar slants^[^^14^^]^. After 24-h incubation on MRS agar plates, the morphological features and Gram staining of the isolated strains were investigated. The activity of catalase and the gas release in glucose medium were also studied.

**Molecular identification of lipase-producing Lactobacilli**

Selected lipase-producing isolates were cultivated in MRS broth at 37 °C for 18 h and were subjected to DNA extraction following the standard procedures^[^^15^^]^. Final DNA concentration was calculated by measuring the absorbance of 1 µL of the sample at 260/280 nm using the NanoDrop 2000 spectrophotometer (Thermo Scientific). The amplification of 16S rDNA was performed using universal primers of 27f (5`-AGAGTTTGACCTCCTCCTGGCTCAG-3`) and 1492r (5`-GGCTACCTTGTTACGACTT-3`). The PCR cycles started with an initial denaturation at 94 °C for 1 min, followed by 35 cycles of annealing at 58 °C for 30 s, elongation at 72 °C for 1 min, and the final elongation step at 72 °C for 10 min. Dideoxy chain termination method was applied to sequence PCR products. The neighbor-joining phylogeny method was used for the reconstruction of the phylogenetic tree in MEGA software version 5.0. We utilized bootstrapping to assess the reliability of phylogenetic reconstructions (1,000 replicates).

**PCR amplification of lipase gene**

A pair of primers was designed based on the DNA sequence of GDSL lipase/esterase homologue of *L. fermentum* extracted from NCBI nucleotide database (https://www.ncbi.nlm.nih.gov/nuccore/AP008937.1).The sequences of the forward and reverse primers used to amplify GDSL lipase/esterase in four selected isolates were 5'-TTGAGCAAGAAAGTATGGCG-3' and 5'-GGTACTCAAAGTTGTTATTTTTC-3', respectively. PCR was performed as described above, except the annealing temperature which changed to 55 °C.

**Construction of expression cassette**

All DNA manipulations were performed according to Sambrook *et al.*^[16]^. To prepare GDSL-like Lipase/ Acylhydrolas gene expression construct, the target gene was PCR amplified using primers 2F5'd (ATATGAATTCTTGAGCAAGAAAGTATGGCG) and 2R5'd (ATATGAGCTCCTACCTCGTTTGGTA CTC) containing *Eco*RI and *Sac*I restriction sites at 5` and 3`ends, respectively (the underlined sequences). The PCR product was analyzed using agarose gel electrophoresis and the desired ~1-kb fragment was excised and purified using Ron's Gel Extraction Kit. The pET-28a(+) plasmid and the lipase gene fragment were then digested with *Eco*RI and *Sac*I, and the digestion products were cleaned and ligated using T4 DNA ligase. The competent *E. coli* DH5α cells were then transformed using the recombinant pET-28a(+) expression vector. The kanamycin-resistant colonies were screened by colony PCR method. A confirmed recombinant plasmid was finally transformed into the *E. coli* BL21 (DE3) for protein expression experiments^[^^17^^]^.

**Protein expression**

A single colony of transformed *E. coli* BL21 (DE3) was grown in the LB medium containing 50 µg/ml of kanamycin at 37 °C. Once OD_600_ approached 0.5, IPTG was added to a final concentration of 1 mM. After 2, 4, 6, and 12 hours of IPTG induction, cells were harvested by centrifugation (at 4000 ×g, at 4 °C for 10 min), and the pellet was then resuspended in 1× PBS with pH 8.0 and sonicated on ice in 10-s pulses for 60 s. The insoluble fraction of the lysate was removed by centrifugation at 12000 ×g at 4 °C for 10 min, and the supernatant was filtered through a 0.2-µm pore size filter and assessed using the SDS-PAGE.

**Recombinant protein purification**

The *E. coli* culture was inoculated into 250 ml of LB medium and incubated at 37 °C for 18h. When the OD_600_ of the medium reached 0.5, IPTG was added to a final concentration of 1 mM. After collecting the cell pellet by centrifugation (8000 ×g, at 4 °C, for 10 min), pellet weights were determined, and the pellets were resuspended in the lysis/wash buffer (50 mM of KH_2_PO_4_, 300 mM of KCl, and 5 mM of imidazole, pH 8.0). The lysate was sonicated on ice (four cycles, 1 min), and supernatant was separated by centrifugation (12000×g, at 4 °C, for 20 min,). The supernatant was filtered through a 0.2-µM filter immediately before applying to the cartridge. Metal afﬁnity chromatography was applied for the purification of the His-tagged target protein from the crude extract. According to manufacturer’s instructions, Ni-NTA (BIO-RAD) resin was previously equilibrated with lysis/wash buffer. Then recombinant protein was eluted with 250 mM of imidazole, and eluted protein was dialyzed against imidazole-free buffer. To detect the recombinant protein in the fractions and determine the purity and apparent molecular weight, the collected fractions were analyzed using SDS-PAGE containing 15% (w/v) polyacrylamide and stained with Coomassie Brilliant Blue. After SDS-PAGE analysis, fractions containing pure lipase were combined, and protein concentration was estimated using Bradford method^[^^18^^]^.

**Enzyme activity assay**

Spectrophotometry at the wavelength of 410 nm was used for quantification of enzymatic activity of the purified recombinant protein using specific substrates of *ρ*-NPA at room temperature for 10 min and *ρ*-NPP at 37 °C for 20 min. For final substrate concentration of 0.25 mM, 5.5 mM of each *ρ*-NPA and *ρ*-NPP stock solution was prepared separately in isopropanol and mixed with a 50-mM Tris-HCl buffer (pH 8.0). Reaction was initiated by adding 100 µg of the enzyme. Blanks (50 mM of KH_2_PO_4_ and 300 mM of KCl, pH 8.0) were used instead of enzyme solution. Overall, 1 ml of isopropanol containing 3 mg of *ρ*-NPA or *ρ*-NPP was mixed with 9 ml of 0.05 M Tris-HCl buffer at pH 8.0, containing 20 mg of sodium deoxycholate, 0.05 mg of Triton X-100, and 10 mg of Gum Arabic. Next, 2.4 ml of the fresh substrate solution was preheated at 37 °C and then mixed with 0.1 ml of the supernatant^[^^19^^]^. The same procedure was also applied using the phosphate buffer. One unit of lipase activity was calculated as the amount of enzyme that releases 1 μmol of *ρ*-nitrophenol per minute. Concentration of *ρ*-nitrophenol was read at 410 nm by using the molar extinction coefficient of 5487.25 M^-1^ cm^-1[^^9^^]^.

## RESULTS

**Isolation and molecular identification of lipase-producing lactobacilli**

From 25 strains, 5 strains producing lipolytic enzymes were detected following the formation of clear haloes around the colonies. The strains were identified based on 16S rDNA sequencing. As depicted in Figure 1, one isolate (5a) was identified as Enterococcus species with 98% similarity. The other four isolates corresponded to *L. fermentum* with 98% similarity*. *All sequences have been deposited in the GeneBank under accession numbers MH333208 (https://www.ncbi.nlm.nih.gov/ nuccore/MH333208), MH333209 (6a), MH333210 (6b) and MH333211 (6c). 

**Fig. 1:**
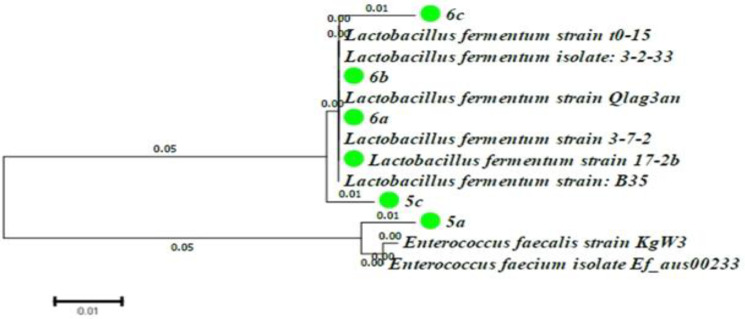
Neighbor-joining phylogenetic tree lipase-producing lactobacillus isolates

The 5c isolate with the highest enzyme activity was selected for further investigation.

**Characterization of GDSL-like Lipase/Acylhydrolas gene**

 The GDSL esterases/Lipases-encoding gene was amplified in 5c isolate genome by specific primers. PCR product was sequenced and then analyzed by BLAST. The GDSL esterases/lipases-encoding gene was 918 base pairs long and encoded a polypeptide of 305 amino acids with a calculated molecular mass of 34342.1 Da and theoretical pI of 9.66. The BLAST analysis of GDSL lipase/esterase-encoding gene revealed 100% query coverage and 90% sequence identity to GDSL esterases/lipases gene in *L. fermentum* IFO3956 (GenBank number AP008937.1)^[^^11^^,^^20^^]^. This new gene was submitted to the DDBJ/EMBL/GenBank databases under the accession number MH469273.1

**Recombinant protein expression and purification**

To assess recombinant lipase expression in *E. coli* BL21 (DE3), a pET28a (+) vector containing the coding sequences for lipase and His tag peptide was constructed. After 2-, 4-, 6-, and 12-h induction with IPTG, an expected protein band of about 34 kDa was observed on SDS-PAGE (Fig. 2). The His6-tagged protein was puriﬁed by metal afﬁnity chromatography. The recombinant enzyme was eluted with 250 mM of imidazole solution. A single protein band of about 34 kDa was detected on SDS-PAGE gels (Fig. 3). This observed molecular weight was comparable to the theoretical molecular weight of the full-length recombinant protein (34.342 kDa). According to the Bradford method, the protein concentration was estimated to be 0.1 mg/ml. 

**Substrate speciﬁcity**

Enzyme activity of the purified recombinant protein was quantified at 410 nm spectrophotometrically. The enzyme activity was measured at 0.6 U/ml and 0.2 U/ml in the presence of *ρ-*NPA(C_2_) and *ρ-*NPP(C_16_), as specific short- and long-chain fatty acid substrates, respectively (Fig. 4).

## DISCUSSION

As stated above, little is known on lipase production from Lactobacillus spp. In this sense, the present research was conducted with the aim of cloning, expression, and purification of a new lipase/esterase enzyme from *L. fermentum*. Herein, cloning and expression of the GDSL lipases-encoding gene from a native Lactobacillus isolate in *E. coli* host resulted in the production of an active lipase enzyme. In a similar research, Brod *et al.*^[^^9^^]^ cloned the esterase-encoding gene from *L. plantarum* ATCC 8014 in the *E. coli* DH5 host and produced the recombinant esterase using the pET14b expression vector. Heterologous expression in bacterial hosts, including *E. coli*, has been studied for many bacterial lipase genes as a strategy to produce high quantities of recombinant lipase in a relatively short amount of time. However, conflicting results have been reported in the literature in this field. For instance, there has been a successful case of cloning, sequencing, and expression of the triacylglycerol lipase gene of *Aspergillus oryzae *in *E. coli *species^[^^21^^]^. However, Xuezheng *et al.*^[^^22^^]^ reported low recombinant lipase activity and lipase accumulation in a number of *E. coli* species. Expression in *E. coli* may require *in vitro* refolding of lipase in the presence of specific natural chaperones and foldases^[^^23^^]^.

**Fig. 1:**
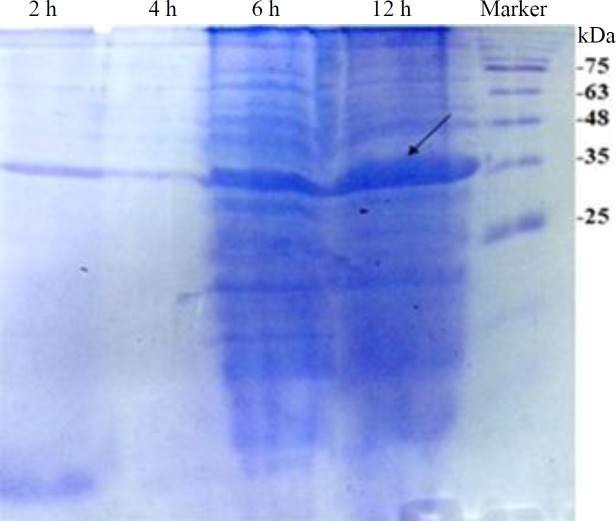
SDS-PAGE analysis of recombinant protein expression in *E. coli* BL21 (DE3) extracted following IPTG induction at different time points (2, 4, 6, and 12 h). IPTG induced the expression of a 34 kDa protein corresponding to the expected molecular weight of recombinant protein. The arrow indicates the position of recombinant protein band

**Fig. 3:**
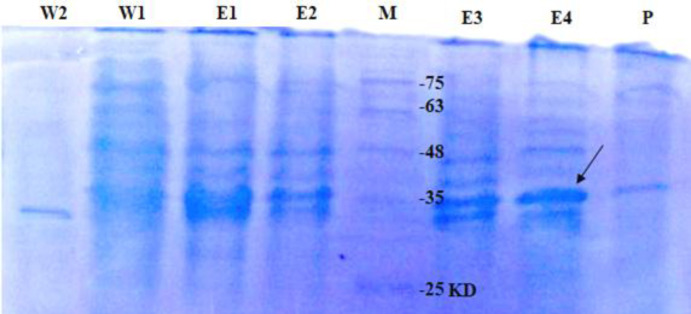
SDS-PAGE analysis of recombinant protein purification fractions by Ni-NTA resin affinity chromatography. After two washing steps (Lanes W1 and W2), recombinant protein was eluted with 250 mM of imidazole solution (lanes E1, E2, E3, and E4). A single protein band with an apparent molecular mass of about 34 kDa was obtained after purification steps (lane P). Lane M is a molecular weight marker. The arrow indicates the position of recombinant protein band

The molecular weight of the most esterases is in the range of 22–60 kDa. The recombinant *L. fermentum* esterase reported in this research generated a single band with an apparent molecular weight of approximately 34 kDa. It is similar to 35-kDa esterase from *L. plantarum*^[^^24^^]^ and is different from the 29-kDa esterase from *L. fermentum*^[^^25^^]^.

**Fig. 4:**
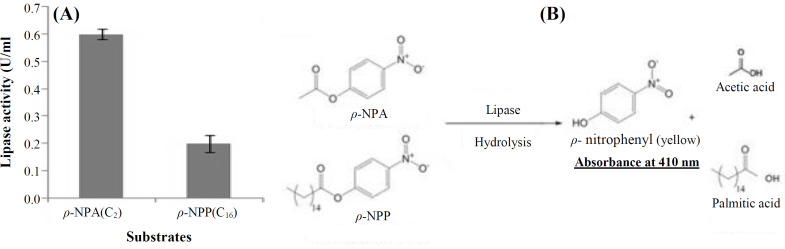
*Substrate specificity of the purified recombinant protein toward two different *ρ*-nitrophenyl esters,* ρ*-NPA(C*_2_*) and *ρ*-NPP(C*_16_*) . (A) Enzyme activity was quantified at 410 nm, pH 8.0,* *at 25 °C. Data were expressed as mean ± SD (n* *=* *3); (B) hydrolysis reaction of ρ-NPA in the presence of lipase*

The estrase enzyme hydrolyzes *p*-nitrophenyl esters in the C2 to C4 range and triacylglycerols lower than C10, and it has no interaction with nitrophenyl esters higher than C6^[^^26^^]^. In this research, the substrate specificity of recombinant esterase from *L. fermentum *showed excellent selectivity for *p*- NP esters of short-chain fatty acids, which agrees with the finding achieved for J15 GDSL esterase from the *Photobacterium *sp. strain J15^[^^6^^]^ and feruloyl esterase from *L. fermentum *NRRL B-1932^[^^25^^]^. 

In this study, a novel lipase/esterase from *L. fermentum*, belonging to lipolytic family, was cloned and expressed. Being multifunctional, the novel lipase/esterase holds great potentials for research purposes given its broad substrate specificity and regiospecificity. Although numerous GDSL esterases and lipases have been detected in bacteria, they are still poorly understood in terms of their structures, functions, and physiological functions. Crude extracellular GDSL esterases/lipases concentrated by ultrafiltration of cell-free culture supernatant of *L. fermentum *showed great activity toward *ρ*-NPA (short-chain fatty acid substrate). Further structural and biochemical characterization of this enzyme is needed to understand its reaction mechanism, which could be the subject matter of future studies.

## DECLARATIONS

### Ethical statement

Not applicable. 

### Data availability 

The data supporting the findings of this study are available on request from the corresponding author.

### Author contributions

SP, investigation, methodology, resources; RK, project administration, methodology, supervision; SG, methodology, supervision, resources; EMQ, investigation, writing original draft, review, editing.

### Conflict of interest

None declared.

### Funding/support

This research was funded by the Department of Biotechnology, Faculty of Biological Sciences, Alzahra University, Tehran, Iran.
